# Screening for Coronary Artery Disease in Patients with Diabetes

**DOI:** 10.1007/s11886-023-01999-z

**Published:** 2023-11-20

**Authors:** Antti Saraste, Juhani Knuuti, Jeroen Bax

**Affiliations:** 1 Heart Center, Turku University Hospital and University of Turku, Hämeentie 11, Turku, 20520 Finland; 2 Turku PET Centre, Turku University Hospital and University of Turku, Turku, Finland; 3 Department of Cardiology, Leiden University Medical Center, Leiden, Netherlands

**Keywords:** Coronary artery disease, Coronary CT angiography, Coronary calcium score, Myocardial perfusion imaging, Screening, Diabetes

## Abstract

**Purpose of Review:**

The study aims to describe methods for detecting subclinical coronary artery disease (CAD) and their potential implications in asymptomatic patients with diabetes.

**Recent Findings:**

Imaging tools can assess non-invasively the presence and severity of CAD, based on myocardial ischemia, coronary artery calcium score, and coronary computed tomography coronary angiography. Subclinical CAD is common in the general population ageing 50 to 64 years with any coronary atherosclerosis present in 42.1% and obstructive CAD in 5.2%. In patients with diabetes, an even higher prevalence has been noted. The presence of myocardial ischemia, obstructive CAD, and the extent of coronary atherosclerosis provide powerful risk stratification regarding the risk of cardiovascular events. However, randomized trials evaluating systematic screening in the general population or patients with diabetes have demonstrated only moderate impact on management and no significant impact on patient outcomes.

**Summary:**

Despite providing improved risk stratification, systematic screening of CAD is not recommended in patients with diabetes.

## Introduction

Coronary artery disease (CAD) is a major cause of mortality and morbidity in patients with diabetes, although the risk of cardiovascular events has decreased over time [1, 2]. A meta-analysis of 102 prospective studies including 698,782 individuals found that diabetes confers a two-fold excess risk of CAD (hazard ratio [HR] 2.0, 95% CI 1.8–2.2) [1]. Studies also show that clinical symptoms of CAD in patients with diabetes are often less severe and atypical in presentation as compared to individuals without diabetes [3]. Current standard of care emphasizes comprehensive management of cardiometabolic risk factors guided by systematic risk stratification based on the age, the number of associated conventional risk factors for cardiovascular disease, diabetes-specific information (type of diabetes, age at diabetes diagnosis, degree of hyperglycemia, and renal function), the presence of atherosclerotic cardiovascular disease, and target organ damage [4]. However, the high cardiovascular risk in diabetes has generated interest for early detection of asymptomatic CAD using screening tests.

The primary purpose of screening for CAD in patients with diabetes would be to improve risk prediction at an individual level and thereby, identify patients whose prognosis could be improved with an intervention (medication for risk factor modification or coronary revascularization). Recent technological advances have produced sophisticated imaging tools to assess non-invasively the presence and severity of CAD, including functional tests for myocardial ischemia, coronary artery calcium (CAC) scan, and coronary computed tomography coronary angiography (CTA) [5]. In asymptomatic individuals, evaluation of the prognostic value, impact on treatment decisions and outcomes, cost–benefit ratio, and potential harms of these imaging tools are particularly important. This focused review aims at presenting imaging methods to detect CAD and their potential implications in asymptomatic patients with diabetes.

## Screening Tests and Prevalence of CAD in Diabetes

Asymptomatic CAD in patients with diabetes includes atherosclerotic lesions that do not cause luminal narrowing to the extent that provokes myocardial ischemia and those obstructive lesions that can produce ischemia during stress testing, but may not be appreciated clinically by the patient. The prevalence of asymptomatic CAD in patients with diabetes depends significantly on the method of screening and what test result is considered diagnostic for CAD. The accuracy of diagnostic tests for obstructive CAD has been summarized in meta-analysis published in 2018 [5]. However, in this meta-analysis, the patients were largely referred for testing because CAD was suspected, whereas the findings may not apply to a screening population.

Exercise ECG in patients with diabetes is attractive due to low cost, simplicity, and wide availability. In asymptomatic patients with diabetes, the sensitivity and specificity of exercise ECG in diagnosing obstructive CAD were 47% and 81%, respectively [6]. Compared with exercise ECG, non-invasive functional imaging tests have superior diagnostic performance for the detection of obstructive CAD [5]. However, an exercise ECG provides complementary clinically useful information beyond ECG changes about symptoms, exercise tolerance, arrhythmias, blood pressure response, and event risk [7, 8]. In 5,783 overweight or obese asymptomatic middle-aged men and women with type 2 diabetes, Curtis et al. found that exercise-induced abnormalities were present in 22.5% of participants, such as impaired exercise capacity in 12% and ST-segment depression in 7.6% [8].

**Fig. 1:**
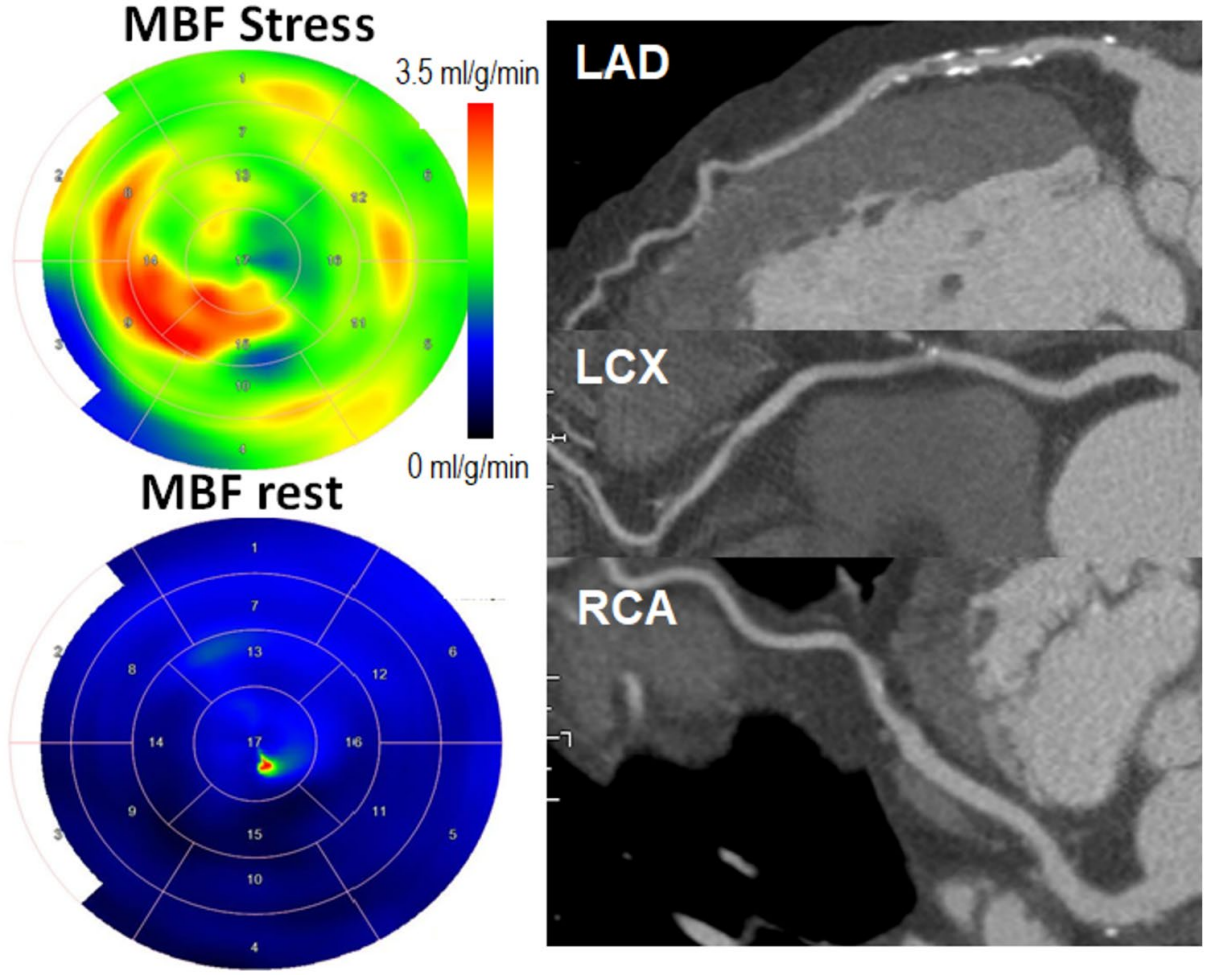
Coronary CT angiography and positron emission tomographic (PET) myocardial perfusion images in a 62-year-old man with atypical chest pain, diabetes, dyslipidemia, and hypertension. Coronary artery calcium (CAC) scan showed moderate CAC score of 210. Coronary CT angiography showed partially calcified atherosclerosis in the left anterior descending, left circumflex, and right coronary arteries (LAD, LCX, and RCA, respectively). Maps of myocardial blood flow (MBF) measured by ^15^O-water PET showed no significant myocardial ischemia and invasive coronary angiography showed no obstructive CAD. However, based on PET, coronary flow reserve was impaired (1.8), suggesting coronary microvascular dysfunction

Non-invasive functional imaging tests for CAD include radionuclide myocardial perfusion imaging with single-photon emission computed tomography (SPECT) or positron emission tomography (PET), stress echocardiography, or stress cardiac magnetic resonance imaging (CMR) (Fig. 1). Detection of obstructive CAD is based on perfusion abnormalities or ischemic wall motion abnormalities provoked by exercise or pharmacological stress. Functional imaging tests detect obstructive CAD with a high sensitivity (85–90%) and specificity (70–85%) [5] and permit the detection of silent myocardial ischemia in patients with diabetes. Patients with type 2 diabetes, who are generally older than patients with type 1 diabetes, more frequently have silent perfusion abnormalities during stress testing with a prevalence of approximately 22% in large, prospective cohorts of asymptomatic high-risk diabetics [9–11]. The prevalence of silent myocardial ischemia appears similar when either radionuclide myocardial perfusion imaging or dobutamine stress echocardiography is used [12]. Similar to the general population, the data in patients with diabetes suggest that routine screening with myocardial perfusion imaging of all asymptomatic patients is likely to have a low yield and a limited effect on patient outcomes. The yield of ischemia testing can be improved by focusing on a high-risk groups, such as patients with symptoms, peripheral vascular disease, carotid plaque, chronic kidney disease, an abnormal ECG, or a high coronary artery calcium score [13, 14].

Coronary CTA is an anatomical imaging modality that visualizes the coronary artery lumen and wall using an intravenous contrast agent (Fig. 1). Thereby, it visualizes the burden of non-obstructive calcified or non-calcified plaques and provides very high sensitivity of 97% for the detection of obstructive CAD defined by invasive coronary angiography [5]. Specificity of coronary CTA is lower than sensitivity, particularly in studies using invasive fractional flow reserve (FFR) rather than angiography as the reference standard (53% and 78%, respectively) [5]. Stenoses estimated to be 50 − 90% by visual inspection are not necessarily functionally significant; that is, they do not always induce myocardial ischemia, and severe calcifications may lead to overestimation of stenosis severity by coronary CTA [7].

Recent studies using coronary CTA have shown that subclinical CAD is common in the general, middle-aged population. In a randomly invited cohort of 25,182 individuals ageing from 50 to 64 years, CTA detected any coronary atherosclerosis in 42.1% and obstructive CAD (≥ 50% stenosis) in 5.2% [15•]. Studies in cohorts of patients with diabetes indicate that the prevalence of subclinical CAD is higher than would be expected in the general population. In a substudy of the CONFIRM registry, which included 400 asymptomatic patients with diabetes and no prior history of CAD, 70% of patients had any coronary atherosclerosis on coronary CTA and 27.8% showed obstructive CAD [16]. However, it is notable that approximately 25 to 30% of asymptomatic diabetic patients have no demonstrable plaque on coronary CTA supporting the concept that diabetes itself is not a CAD equivalent [16–18].

Measurement of CAC score by non-contrast cardiac CT is an inexpensive, low-radiation imaging method to detect and quantify coronary artery atherosclerotic plaque burden. Measurement of CAC score is an effective marker to refine cardiovascular risk stratification in an asymptomatic population [19, 20]. Across age groups, asymptomatic adults with diabetes have higher median CAC scores than individuals without diabetes [21–23]. However, a meta-analysis of eight studies (6521 patients) revealed that 28.5% had a CAC score < 10, indicating that nearly 3 in 10 patients had very little or no calcified coronary artery atherosclerosis [24]. Importantly, CAC scoring does not allow differentiation between non-obstructive and obstructive CAD. However, the extent of CAC has been shown to be associated with the prevalence of inducible ischemia by SPECT myocardial perfusion imaging [25]. The CAC score threshold at which the prevalence of ischemia increases substantially is > 400 with 7% and 15% of asymptomatic non-diabetic individuals showing ischemia if CAC score was > 400 and > 1000, respectively [25]. In patients with metabolic syndrome or diabetes, a high prevalence of ischemia (13%) has been noted even among patients with CAC scores of 100–399 [26].

In summary, with the exception of exercise ECG, all non-invasive diagnostic tests for CAD have demonstrated very high sensitivity and specificity for detecting obstructive CAD. Despite higher prevalence of coronary atherosclerosis and obstructive CAD in patients with diabetes as compared to the general population, studies using coronary CTA and CAC scan have shown that a relatively large proportion of patients with diabetes has no or only minimal coronary atherosclerosis, which may have implications for intensity of preventive therapies.

## Screening Tests of CAD and Prognosis

All non-invasive diagnostic tests for obstructive CAD predict cardiac events based on the severity of their findings, also in patients with diabetes mellitus [7, 14]. A meta-analysis of prospective cohorts found a 3.5-fold increased risk of cardiac events associated with silent myocardial ischemia [27]. In the general population, a normal myocardial ischemia test is associated with a low risk (< 1% per year) of cardiac events [28]. However, the elevated cardiovascular risk in the diabetic patient alters the post-test risk assessment. In a meta-analysis of 14 studies involving 13,493 diabetic patients, a normal myocardial perfusion scan was associated with a slightly higher risk of adverse cardiac events in diabetic (1.6% per year) than non-diabetic patients (< 1% per year) [29]. In 5456 patients undergoing stress echocardiography of whom 749 had diabetes, diabetic patients had a 2.5-fold greater annual event rate than non-diabetic patients during a median follow-up of 31 months [30]. Notably, the warranty period of a normal myocardial ischemia test appears to be shorter in diabetic patients, with event-free survival curves beginning to diverge from non-diabetic populations in the second year after the index normal scan [13, 30, 31]. In 563 diabetic patients, there were no events in the first 2 years of follow-up among patients with a normal stress echocardiography, but at 3 and 5 years, the event rate for the normal group was 2% and 8%, respectively [30].

The non-invasive assessment of coronary flow reserve (CFR) using PET integrates the effects of focal coronary stenosis, diffuse disease, and coronary microvascular function [32]. A progressive decline in CFR from insulin resistance to diabetes has been described [33]. Reduced CFR has been found as a strong prognostic marker in patients with diabetes [34, 35]. A study by Murthy et al. found an equivalent and low cardiac mortality risk between diabetic patients without known CAD but with CFR > 1.6 and those without diabetes [34]. In contrast, the subgroup of diabetic patients without known CAD but with CFR < 1.6 had essentially the same risk as patients without diabetes but with CAD [34].

The prognostic value of coronary CTA findings in asymptomatic individuals was evaluated in a prospective observational study where the findings remained clinically blinded, thus reflecting the natural history of subclinical coronary atherosclerosis [36]. In 9533 asymptomatic, mostly non-diabetic individuals aged 40 years or older, obstructive and extensive (atherosclerosis in > 5 coronary segments) disease was associated with a 12-fold increased risk of myocardial infarction as compared to those with normal coronary arteries, despite adjustment for age, sex, known clinical risk factors, and concomitant therapies during a median of 3.5-year follow-up [36]. Similar to non-diabetic patients, the absence of atherosclerosis by coronary CTA is associated with very low risk of cardiovascular events [16, 17]. A meta-analysis of eight studies involving 6225 diabetic patients who underwent coronary CTA due to suspected CAD found an annualized event rate of 0.1% in the absence of any CAD [17].

Increased CAC in persons with metabolic syndrome and diabetes is associated with increased cardiovascular events [13, 37] and mortality [38, 39]. CAC was a better predictor of incident cardiovascular events compared with the Framingham risk score and the UKPDS (United Kingdom Prospective Diabetes Study) (area under the curve 0.76, 0.70, and 0.69, respectively) and it improved classification of risk [37]. In a population of 9715 individuals including 810 diabetics, the absence of CAC predicted a low short-term risk of death (2.6% at 5 years) for diabetic patients, which was slightly higher, but statistically similar to that of non-diabetic patients [39]. In an observational study of 2384 patients with diabetes CAC allowed identification of patients at lower risk for whom aspirin preventive treatment might not be beneficial [40].

## Does Screening for Subclinical CAD Improve Outcomes?

Five prospective randomized trials have evaluated the impact of routine screening for subclinical CAD on outcomes of asymptomatic patients with type 2 diabetes [11, 41–44].

In a study of 1123 asymptomatic patients with type 2 diabetes conducted between 2000 and 2007 in the USA, the DIAD investigators randomized patients with a normal resting ECG and no clinical evidence of CAD to either screening with adenosine-stress radionuclide myocardial perfusion imaging or no screening [9, 42]. The prevalence of silent myocardial ischemia was 22%, with high-risk imaging results (defined as perfusion defects of at least 5% of the myocardium) in 6%. However, there were no significant differences with regard to medical treatment and the rate of revascularization between groups. After a mean follow-up of 4.8 years, there was no significant difference in the primary endpoint (cardiac death or myocardial infarction) between the screening and no-screening groups (2.7 versus 3.0%, respectively).

In a study of 631 asymptomatic patients with type 2 diabetes and at least two other cardiovascular risk factors conducted between 2000 and 2005 in France, the DYNAMIT investigators randomized patients to either screening of myocardial ischemia, primarily by exercise ECG, or no screening [11]. In the screened group, the prevalence of silent myocardial ischemia was 21.5%. After a mean follow-up of 3.5 years, there was no significant difference in the composite primary endpoint (all-cause mortality, myocardial infarction, stroke, or heart failure requiring emergency intervention) between the screening group and no-screening group (2.6 versus 2.4% annually; adjusted hazard ratio [HR] 1.0, 95% CI 0.6–1.7).

The FACTOR-64 trial, conducted between 2007 and 2014 in the USA, included 900 asymptomatic patients with type 1 or 2 diabetes who were randomized to coronary CTA screening or no screening [43]. Specific treatment targets for cholesterol, blood glucose, and blood pressure were set based on the CTA results. Furthermore, patients with obstructive CAD were sent for further non-invasive testing or invasive coronary angiography. Among patients randomized to CTA screening, the prevalence of mild, moderate, and severe CAD was 31, 46, and 12%, respectively. After a mean follow-up of 4 years, there was no significant difference in the primary endpoint (all-cause mortality, myocardial infarction, or unstable angina) following screening with coronary CTA or no screening (6.2 versus 7.6%, HR 0.8, 95% CI 0.5–1.3). Furthermore, differences in risk factor levels between both groups were modest after treatment. Coronary angiography and revascularization rates were slightly higher in the screening arm compared with the standard-of-care arm.

The DADDY-D trial, conducted between 2007 and 2012 in a single center in Italy, randomized 520 patients with type 2 diabetes without known CAD and high cardiovascular risk score to screening with exercise ECG versus no screening [44]. Silent myocardial ischemia was documented in 20 (7.6%) patients and coronary revascularization was performed in 12 (4.6%). Over a median follow-up of 3.6 years, there were no differences in the occurrence of cardiovascular death, myocardial infarction, or the combination of both endpoints.

The randomized controlled trials above may have been underpowered to detect effects of screening due to low rate of events (annual rate of major cardiac events < 1%). Data from a meta-analysis of these five trials including total of 3299 patients with diabetes found that non-invasive CAD screening significantly reduced the rate of any cardiac event (cardiac death, non-fatal myocardial infarction, unstable angina, or heart failure hospitalization) by 27% (RR 0.73; 95% CI, 0.55–0.97; *P* = 0.028), driven by non-significant reductions in non-fatal myocardial infarction (RR 0.65; *P* = 0.062) and hospitalization for heart failure (RR 0.61; *P* = 0.1) [45]. There was no difference in cardiac death between screening and no screening (RR 0.92; 95% CI, 0.53–1.60; *P* = 0.77). In line with this, in a recently published randomized controlled trial involving 46,611 men aged 65–74 years, routine cardiovascular screening for CAC, aortic aneurysm, atrial fibrillation, peripheral artery disease, hypercholesterolemia, diabetes, and hypertension did not significantly reduce the incidence of death from any cause after a median follow-up of 5.6 years (HR 0.95; 95% CI, 0.90–1.0, *P* = 0.06), also in a pre-specified diabetes subgroup [46••]. However, the study suggested a greater benefit in a subgroup of patients ageing < 70 years [46••]. Finally, The BARDOT trial prospectively recruited 400 asymptomatic patients with type 2 diabetes and randomized those with myocardial ischemia to revascularization or medical therapy [10]. Although patients randomized to revascularization showed lower rate of asymptomatic CAD progression (myocardial ischemia or new scar) on follow-up imaging (54.3% vs. 15.8%; *P* < 0.001) after 2 years, rates of major adverse cardiovascular events (cardiac death, myocardial infarction, or symptom-driven revascularization) were similar (*P* = 0.215) between groups [10].

## Practical Implications and Future Directions

European and American societies do not recommend systematic screening of CAD in asymptomatic diabetic patients [4, 47], based on the negative results of the aforementioned screening trials. Despite improved risk stratification, studies have not shown outcome benefit from systematic screening for subclinical CAD in diabetes. Furthermore, as long as cardiovascular risk factors are treated, the results of screening would have relatively limited impact on medical therapy in diabetic patients, since aggressive preventive measures, such as control of blood pressure and lipids, would already be indicated. Indeed, in the aforementioned studies, the impact of imaging findings on risk factor modification was modest. Further large and appropriately powered trials are required to allow a more precise analysis of the magnitude of benefit and to assess pre-specified subgroups in which screening strategies may offer larger benefits. Ongoing clinical trials of targeting preventative therapies in persons screened for subclinical CAD, such as the DANE-HEART (Computed Tomography Coronary Angiography for Primary Prevention; ClinicalTrials.gov: NCT05677386) and SCOT-HEART 2 (Computed Tomography Coronary Angiography for the Prevention of Myocardial Infarction; ClinicalTrials.gov: NCT03920176) trials, are expected to provide more insights in this topic. Then, cost effectiveness studies should assess the financial impact and economic benefits of a CAD screening program in diabetic patients.

## Conclusions

Detection of subclinical CAD can provide improved risk stratification in asymptomatic patients with diabetes, but outcome studies do not support systematic screening for CAD in diabetes.

## Data Availability

No new data were generated or analysed in support of this research.

## References

[1] Sarwar N, Gao P, Seshasai SR, Gobin R, Kaptoge S, Di Angelantonio E (2010). Diabetes mellitus, fasting blood glucose concentration, and risk of vascular disease: a collaborative meta-analysis of 102 prospective studies. Lancet.

[2] Sattar N, McMurray J, Borén J, Rawshani A, Omerovic E, Berg N (2023). Twenty years of cardiovascular complications and risk factors in patients with type 2 diabetes: a nationwide Swedish cohort study. Circulation.

[3] Krishnaswami A, Hardison R, Nesto RW, Sobel B, BARI 2D Investigators (2012). Presentation in patients with angiographically documented coronary artery disease and type II diabetes mellitus (from the BARI 2D Clinical Trial). Am J Cardiol.

[4] Marx N, Federici M, Schütt K, Müller-Wieland D, Ajjan RA, Antunes MJ (2023). 2023 ESC Guidelines for the management of cardiovascular disease in patients with diabetes. Eur Heart J.

[5] Knuuti J, Ballo H, Juarez-Orozco LE, Saraste A, Kolh P, Rutjes AWS (2018). The performance of non-invasive tests to rule-in and rule-out significant coronary artery stenosis in patients with stable angina: a meta-analysis focused on post-test disease probability. Eur Heart J.

[6] Lee DP, Fearon WF, Froelicher VF (2001). Clinical utility of the exercise ECG in patients with diabetes and chest pain. Chest.

[7] Knuuti J, Wijns W, Saraste A, Capodanno D, Barbato E, Funck-Brentano C (2020). 2019 ESC guidelines on the diagnosis and management of chronic coronary syndromes: the Task Force for diagnosis and management of chronic coronary syndromes of the European Society of Cardiology (ESC). Eur Heart J.

[8] Curtis JM, Horton ES, Bahnson J, Gregg EW, Jakicic JM, Regensteiner JG (2010). Prevalence and predictors of abnormal cardiovascular responses to exercise testing among individuals with type 2 diabetes: the Look AHEAD (Action for Health in Diabetes) study. Diabetes Care.

[9] Wackers FJ, Young LH, Inzucchi SE (2004). Detection of silent myocardial ischemia in asymptomatic diabetic subjects: the DIAD study. Diabetes Care.

[10] Zellweger MJ, Maraun M, Osterhues HH, Keller U, Muller-Brand J, Jeger R (2014). Progression to overt or silent CAD in asymptomatic patients with diabetes mellitus at high coronary risk: main findings of the prospective multicenter BARDOT trial with a pilot randomized treatment substudy. JACC Cardiovasc Imaging.

[11] Lièvre MM, Moulin P, Thivolet C, Rodier M, Rigalleau V, Penfornis A (2011). Detection of silent myocardial ischemia in asymptomatic patients with diabetes: results of a randomized trial and meta-analysis assessing the effectiveness of systematic screening. Trials.

[12] Jacqueminet S, Barthelemy O, Rouzet F, Isnard R, Halbron M, Bouzamondo A (2010). A randomized study comparing isotope and echocardiography stress testing in the screening of silent myocardial ischaemia in type 2 diabetic patients. Diabetes Metab.

[13] Anand DV, Lim E, Hopkins D (2006). Risk stratification in uncomplicated type 2 diabetes: prospective evaluation of the combined use of coronary artery calcium imaging and selective myocardial perfusion scintigraphy. Eur Heart J.

[14] Budoff MJ, Raggi P, Beller GA, Berman DS, Druz RS, Malik S (2016). Noninvasive cardiovascular risk assessment of the asymptomatic diabetic patient: the Imaging Council of the American College of Cardiology. JACC Cardiovasc Imaging.

[15] Bergström G, Persson M, Adiels M, Björnson E, Bonander C, Ahlström H (2021). Prevalence of subclinical coronary artery atherosclerosis in the general population. Circulation.

[16] Min JK, Labounty TM, Gomez MJ, Achenbach S, Al-Mallah M, Budoff MJ (2014). Incremental prognostic value of coronary computed tomographic angiography over coronary artery calcium score for risk prediction of major adverse cardiac events in asymptomatic diabetic individuals. Atherosclerosis.

[17] Celeng C, Maurovich-Horvat P, Ghoshhajra BB (2016). Prognostic value of coronary computed tomography angiography in patients with diabetes: a meta-analysis. Diabetes Care.

[18] Rana JS, Dunning A, Achenbach S, Al-Mallah M, Budoff MJ, Cademartiri F (2012). Differences in prevalence, extent, severity, and prognosis of coronary artery disease among patients with and without diabetes undergoing coronary computedtomography angiography: results from 10,110 individuals from the CONFIRM (COronary CT Angiography EvaluatioN ForClinical Outcomes): an InteRnational Multicenter Registry. Diabetes Care.

[19] Erbel R, Mohlenkamp S, Moebus S, Schmermund A, Lehmann N, Stang A (2010). Coronary risk stratification, discrimination, and reclassification improvement based on quantification of subclinical coronary atherosclerosis: the Heinz Nixdorf Recall study. J Am Coll Cardiol.

[20] Polonsky TS, McClelland RL, Jorgensen NW, Bild DE, Burke GL, Guerci AD (2010). Coronary artery calcium score and risk classification for coronary heart disease prediction. JAMA.

[21] Wong ND, Nelson JC, Granston T, Bertoni AG, Blumenthal RS, Carr JJ (2012). Metabolic syndrome, diabetes, and incidence and progression of coronary calcium: the Multiethnic Study of Atherosclerosis study. JACC Cardiovasc Imaging.

[22] Hoff JA, Quinn L, Sevrukov A, Lipton RB, Daviglus M, Garside DB (2003). The prevalence of coronary artery calcium among diabetic individuals without known coronary artery disease. J Am Coll Cardiol.

[23] Dabelea D, Kinney G, Snell-Bergeon JK, Hokanson JE, Eckel RH, Ehrlich J (2003). Effect of type 1 diabetes on the gender difference in coronary artery calcification: a role for insulin resistance? The Coronary Artery Calcification in Type 1 Diabetes (CACTI) Study. Diabetes.

[24] Kramer CK, Zinman B, Gross JL, Canani LH, Rodrigues TC, Azevedo MJ (2013). Coronary artery calcium score prediction of all cause mortality and cardiovascular events in people with type 2 diabetes: systematic review and meta-analysis. BMJ.

[25] Berman DS, Wong ND, Gransar H, Miranda-Peats R, Dahlbeck J, Hayes SW (2004). Relationship between stress-induced myocardial ischemia and atherosclerosis measured by coronary calcium tomography. J Am Coll Cardiol.

[26] Wong ND, Rozanski A, Gransar H, Miranda-Peats R, Kang X, Hayes S (2005). Metabolic syndrome and diabetes are associated with an increased likelihood of inducible myocardial ischemia among patients with subclinical atherosclerosis. Diabetes Care.

[27] Zhang L, Li H, Zhang S, Jaacks LM, Li Y, Ji L (2014). Silent myocardial ischemia detected by single photon emission computed tomography (SPECT) and risk of cardiac events among asymptomatic patients with type 2 diabetes: a meta-analysis of prospective studies. J Diabetes Complications.

[28] Smulders MW, Jaarsma C, Nelemans PJ, Bekkers SCAM, Bucerius J, Leiner T (2017). Comparison of the prognostic value of negative non-invasive cardiac investigations in patients with suspected or known coronary artery disease—a meta-analysis. Eur Heart J Cardiovasc Imaging.

[29] Acampa W, Cantoni V, Green R, Maio F, Daniele S, Nappi C (2014). Prognostic value of normal stress myocardial perfusion imaging in diabetic patients: a meta-analysis. J Nucl Cardiol.

[30] Elhendy A, Adelaide AM, Mahoney DW, Pellikka PA (2001). Prognostic stratification of diabetic patients by exercise echocardiography. J Am Coll Cardiol.

[31] Cortigiani L, Bigi R, Sicari R, Landi P, Bovenzi F, Picano E (2006). Prognostic value of pharmacological stress echocardiography in diabetic and nondiabetic patients with known or suspected coronary artery disease. J Am Coll Cardiol.

[32] Taqueti VR, Di Carli MF (2018). Coronary microvascular disease pathogenic mechanisms and therapeutic options: JACC state-of-the-art review. J Am Coll Cardiol.

[33] Prior JO, Quinones MJ, Hernandez-Pampaloni M, Facta AD, Schindler TH, Sayre JW (2005). Coronary circulatory dysfunction in insulin resistance, impaired glucose tolerance, and type 2 diabetes mellitus. Circulation.

[34] Murthy VL, Masano N, Foster CR (2012). Association between coronary vascular dysfunction and cardiac mortality in patients with and without diabetes mellitus. Circulation.

[35] Aljizeeri A, Ahmed AI, Suliman I, Alfaris MA, Elneama A, Al-Mallah MH (2023). Incremental prognostic value of positron emission tomography-derived myocardial flow reserve in patients with and without diabetes mellitus. Eur Heart J Cardiovasc Imaging.

[36] Fuchs A, Kühl JT, Sigvardsen PE (2023). Subclinical coronary atherosclerosis and risk for myocardial infarction in a Danish cohort. Ann Intern Med.

[37] Yeboah J, Erbel R, Delaney JC, Nance R, Guo M, Bertoni AG (2014). Development of a new diabetes risk prediction tool for incident coronary heart disease events: the Multi-Ethnic Study of Atherosclerosis and the Heinz Nixdorf Recall Study. Atherosclerosis.

[38] Raggi P, Shaw LJ, Berman DS, Callister TQ (2004). Prognostic value of coronary artery calcium screening in subjects with and without diabetes. J Am Coll Cardiol.

[39] Valenti V, Hartaigh BO, Cho I, Schulman-Marcus J, Gransar H, Heo R (2016). Absence of coronary artery calcium identifies asymptomatic diabetic individuals at low near-term but not long-term risk of mortality: a 15-year follow-up study of 9715 patients. Circ Cardiovasc Imaging.

[40] Silverman MG, Blaha MJ, Budoff MJ (2012). Potential implications of coronary artery calcium testing for guiding aspirin use among asymptomatic individuals with diabetes. Diabetes Care.

[41] Faglia E, Manuela M, Antonella Q, Michela G, Vincenzo C, Maurizio C (2005). Risk reduction of cardiac events by screening of unknown asymptomatic coronary artery disease in subjects with type 2 diabetes mellitus at high cardiovascular risk: an open-label randomized pilot study. Am Heart J.

[42] Young LH, Wackers FJ, Chyun DA (2009). Cardiac outcomes after screening for asymptomatic coronary artery disease in patients with type 2 diabetes: the DIAD study: a randomized controlled trial. JAMA.

[43] Muhlestein JB, Lappé DL, Lima JA (2014). Effect of screening for coronary artery disease using CT angiography on mortality and cardiac events in high-risk patients with diabetes: the FACTOR-64 randomized clinical trial. JAMA.

[44] Turrini F, Scarlini S, Mannucci C (2015). Does coronary Atherosclerosis Deserve to be Diagnosed earlY in Diabetic patients? The DADDY-D trial. Screening diabetic patients for unknown coronary disease. Eur J Intern Med.

[45] Clerc OF, Fuchs TA, Stehli J, Benz DC, Gräni C, Messerli M (2018). Non-invasive screening for coronary artery disease in asymptomatic diabetic patients: a systematic review and meta-analysis of randomised controlled trials. Eur Heart J Cardiovasc Imaging.

[46] Lindholt JS, Søgaard R, Rasmussen LM, Mejldal A, Lambrechtsen J, Steffensen FH (2022). Five-year outcomes of the Danish Cardiovascular Screening (DANCAVAS) trial. N Engl J Med.

[47] ElSayed NA, Aleppo G, Aroda VR, Bannuru RR, Brown FM, Bruemmer D (2023). Cardiovascular disease and risk management: standards of care in diabetes—2023. Diabetes Care.

